# Depolarization of Hippocampal Neurons Induces Formation of Nonsynaptic NMDA Receptor Islands Resembling Nascent Postsynaptic Densities^1^,^2^,^3^

**DOI:** 10.1523/ENEURO.0066-15.2015

**Published:** 2015-12-08

**Authors:** Jung-Hwa Tao-Cheng, Rita Azzam, Virginia Crocker, Christine A. Winters, Tom Reese

**Affiliations:** 1 Electron Microscopy Facility, National Institute of Neurological Diseases and Stroke, National Institutes of Health, Bethesda, Maryland 20892; 2 Laboratory of Neurobiology, National Institute of Neurological Diseases and Stroke, National Institutes of Health, Bethesda, Maryland 20892

**Keywords:** electron microscopy, extrasynaptic, NMDA receptors, postsynaptic density, synapse formation

## Abstract

Depolarization of neurons in 3-week-old rat hippocampal cultures promotes a rapid increase in the density of surface NMDA receptors (NRs), accompanied by transient formation of nonsynaptic NMDA receptor clusters or NR islands. Islands exhibit cytoplasmic dense material resembling that at postsynaptic densities (PSDs), and contain typical PSD components, including MAGUKS (membrane-associated guanylate kinases), GKAP, Shank, Homer, and CaMKII detected by pre-embedding immunogold electron microscopy. In contrast to mature PSDs, islands contain more NMDA than AMPA receptors, and more SAP102 than PSD-95, features that are shared with nascent PSDs in developing synapses. Islands do not appear to be exocytosed or endocytosed directly as preformed packages because neurons lacked intracellular vacuoles containing island-like structures. Islands form and disassemble upon depolarization of neurons on a time scale of 2-3 min, perhaps representing an initial stage in synaptogenesis.

## Significance Statement

Islands of extrasynaptic NMDA receptors populate the plasma membranes of hippocampal neurons. The receptor islands also contain many typical postsynaptic density (PSD) proteins and thus resemble nascent PSDs. NMDA receptors appear to be exocytosed only individually or in small groups rather than in concentrated clusters, so islands must form by clustering of individual NMDA receptors already in the neuronal plasma membrane. Additional islands rapidly form and resolve when neurons are depolarized during a 2-3 min window. These findings provide possible insight into one of the mechanisms of synapse formation.

## Introduction

Two types of glutamate receptors [AMPA and NMDA receptors (NRs)] support most excitatory synaptic transmission at mammalian CNS synapses. It is well established that AMPA receptors undergo dynamic, activity-dependent changes in distribution at synapses (Anggono and Huganir, 2012). More recently, activity-dependent plasticity of synaptic (Lau and Zukin, 2007; Rebola et al, 2010; Dupuis et al., 2014) as well as extrasynaptic NRs (Rao and Craig, 1997) has been recognized.

Extrasynaptic NRs carry functional implications different from synaptic ones (Hardingham and Bading, 2010; Papouin and Oliet, 2014), and often form distinctive clusters (Petralia et al., 2010). Based on examination of brain by electron microscopy (EM), these clusters appear as “free postsynaptic densities” (PSDs), “nonsynaptic surface specializations,” or “bare densities” (Blue and Parnavelas, 1983; Steward and Falk, 1986; Fiala et al., 1998). Extrasynaptic NR clusters also label for SAP102 and PSD-95 (Sans et al., 2000), two scaffold proteins that are associated with the PSD. The present study focuses on the extrasynaptic clusters of NMDA receptors, which we refer to as nonsynaptic NR islands, in dissociated hippocampal neuronal cultures where experimental conditions can be easily manipulated. To determine whether neuronal activity affects the formation of NR islands, the numbers of islands are compared under control, high K^+^, and recovery conditions, showing the time course of their formation and disassembly. We also looked for associated evidence of exocytosis and endocytosis, which is indicative of trafficking of NMDA receptors.

Because NR islands include a cytoplasmic dense material resembling that at the PSD, we analyzed their composition by EM immunolabeling for seven PSD-associated proteins and found that many of these proteins localized to NR islands. The PSD complex is composed of a network of specialized proteins that are involved in signal transmission and modulation (Sheng and Kim, 2011). These proteins have a layered distribution within PSDs in mature synapses (Valtschanoff and Weinberg, 2001) as well as during development (Petralia et al., 2005). In order to determine the degree of similarity between NR islands and PSDs, labeling frequencies, intensities, and the laminar distribution of PSD proteins at islands were compared with those at PSDs.

## Materials and Methods

### Antibodies

Mouse monoclonal antibodies against NR2B (clone N59/36, at 1:100), SAP102 (clone N19/2 at 1:50), GKAP (clone N127/31 at 1:100), and Shank2 (clone N23B/6 at 1:200) were from NeuroMab; mouse monoclonal antibody against NR1 (clone R1JHL at 1:100) was from Calbiochem; rabbit polyclonal antibody against NR2A/B (catalog #AB1548, at 1:100) was from Chemicon; mouse monoclonal antibodies against GluR2 (clone 6C4 at 1:100) and α-calcium/calmodulin-dependent protein kinase II [CaMKII; clone 6G9(2) at 1:100] were from Millipore; rabbit polyclonal antibody raised to residues 290–307 of PSD-95 (at 1:500) was custom made by New England Peptide; and mouse monoclonal antibody against Homer 1 (clone 2G8, at 1:200) was from Synaptic Systems.

### Dissociated hippocampal neuronal cultures and treatments

The animal protocol was approved by the National Institutes of Health (NIH) Animal Use and Care Committee and conforms to NIH guidelines. Hippocampal cells from 21-d-old embryonic Sprague-Dawley rats of either sex were dissociated and grown on a feeder layer of glial cells for 19–21 d. During experiments (exp), culture dishes were placed on a floating platform in a water bath maintained at 37˚C. The control incubation medium contained the following: 124 mm NaCl, 2 mm KCl, 1.24 mm KH_2_PO_4_, 1.3 mm MgCl_2_, 2.5 mm CaCl_2_, and 30 mm glucose in 25 mm HEPES at pH 7.4. The high K^+^ solution contained 90 mm KCl with osmolarity compensated for by reducing the concentration of NaCl in the control medium. Cell cultures were washed with control medium and treated for 2 min with control or high K^+^ media. For recovery experiments, samples were treated with high K^+^ for 2 min and then washed with control medium (five times within 2 min), then either fixed at 2-3 min after washout of high K^+^ or left in control medium for 30 min. One set of sister cultures treated with different conditions is counted as one experiment. Cells were fixed with 4% paraformaldehyde (EMS) in PBS for 30-45 min and were thoroughly washed before immunolabeling.

### Pre-embedding immunogold labeling and electron microscopy

Fixed samples were washed, and most were then blocked and made permeable with 5% normal goat serum and 0.1% saponin in PBS for 30-60 min. Some samples for labeling with the NR1, NR2B, and GluR2 antibodies were permeabilized with 50% ethanol for 10 min, and then were treated with 5% normal goat serum in PBS for 20-30 min. All steps were performed at room temperature unless otherwise indicated. Samples were incubated with primary and secondary antibodies (Nanogold, Nanoprobes) for 1 h, fixed with 2% glutaraldehyde in PBS for 30 min, then held at 4˚C, washed in water, and silver enhanced (HQ Kit, Nanoprobes), treated with 0.2% osmium tetroxide in 0.1 m phosphate buffer at pH 7.4 for 30 min on ice, en bloc stained with 0.25-0.5% uranyl acetate in acetate buffer at pH 5.0 for 1 h at 4˚C, dehydrated in graded ethanols, and embedded in epoxy resin. Controls for immunolabeling include omitting the primary antibody or comparison between different primary antibodies for specific labeling for different structural entities.

### Sampling of synapses, nonsynaptic NMDA receptor islands, and morphometry

Asymmetric synapses were identified by clusters of synaptic vesicles in the presynaptic terminal, the dense material underneath the postsynaptic membrane, and by the rigidly apposed presynaptic and postsynaptic membranes forming a synaptic cleft. Every synaptic profile encountered within randomly selected grid openings was photographed with a bottom-mounted digital CCD camera which collects 2.6 × 2.6 thousand pixels (XR-100, AMT). Only cross-sectioned synaptic profiles with clearly delineated postsynaptic membranes were included for measurement. There was no difference in the structure or pattern of labeling by various antibodies between dendritic and somal asymmetric synapses, so measurements were pooled from synapses located on dendrites and somas. Since there were many more dendritic than somal asymmetric synapses, the great majority of synapses sampled here were dendritic. To measure labeling intensity at the PSD, every gold particle that lay within the PSD complex was counted. PSD complexes were outlined by the postsynaptic membrane, two parallel lines dropped perpendicular to the postsynaptic membrane, and an arbitrary border 120 nm deep to the postsynaptic membrane (Tao-Cheng et al., 2015). The total amount of label within this designated PSD complex was then divided by the length of the PSD as an index of labeling intensity (number of particles/micrometer length of PSD).

**Figure 1.**
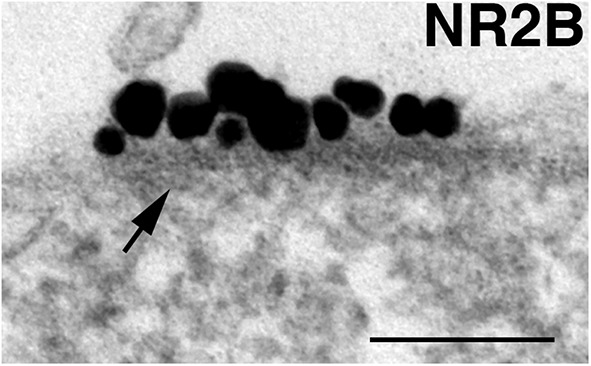
NR island. NR islands are defined by patches of somal/dendritic plasma membrane that label for NMDA receptors (NR2B), and are associated with cytoplasmic dense material (arrow). Scale bar, 0.1 µm.

Nonsynaptic NMDA receptor islands are defined as patches of neuronal plasma membrane at nonsynaptic locations showing clusters of label for NMDA receptors and displaying a characteristic dense material on the cytoplasmic side of the membrane (Fig. 1).

Islands may be apposed by other cellular processes such as glia, dendrites, or axons (Petralia et al., 2010). However, the presence of an apposing axon could indicate a nascent synapse in formation, and the presence of other cellular processes may indicate interactions between the two elements. Thus, in order to study the formation of these islands without the influence of other cellular elements, we limited the sampling of islands to those that lacked any apposing elements.

In dissociated hippocampal cultures, neuronal somas are typically plump in shape and situated on top of a layer of glia. The glia cells are flat and spread out on the substrate, with their cellular processes intermingled with axons and dendrites. Thus, the surface of neuronal somas facing the culture medium is typically not covered by cellular elements. Thin sections were cut en face near these unapposed surfaces of neuronal somas. No distinction in NMDA receptor distribution between the neuronal somal and dendritic plasma membrane was apparent. However, because multiple dendrites can arise from one soma, we limited the sampling of labeling density to somas to ensure that each data point was from a different neuron. Every unapposed neuronal somal profile encountered was photographed for measuring the overall labeling intensity of NMDA receptors. The total number of gold particles on somal plasma membrane, including those clustered in islands, was measured and divided by the length of the plasma membrane.

For the same statistical rationale, islands for quantitation were also sampled only from somas. The frequency of islands was measured in two different ways, and the particular method is specified in each figure or table, as follows: (1) the total number of islands was pooled and divided by the pooled total length of somal plasma membrane to arrive at the number of islands/100 µm plasma membrane; and (2) the total number of islands and the total number of somal profiles were counted, and the final number normalized to express the number of islands per 100 somas. After verifying that the two methods yielded comparable results, the majority of the data were analyzed by the second method because it is more efficient to gather a large data set without photographing the entire length of plasma membrane of every neuronal soma encountered.

**Figure 2.**
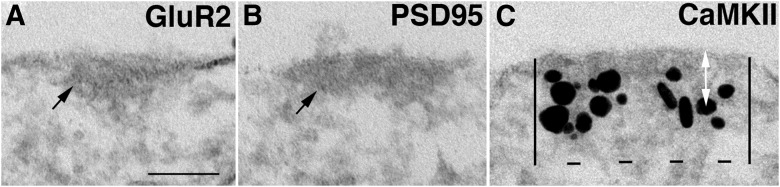
Morphometry of islands. ***A***, ***B***, Islands can be identified by their characteristic cytoplasmic density (***A***, ***B***, arrows) without immunogold labeling. Many islands are not labeled with GluR2 (***A***) or PSD-95 (***B***). ***C***, To measure the label that is located on the cytoplasmic side of islands (CaMKII), two vertical lines extending 120 nm into the cytoplasm are drawn to mark the area for measurement as in the case of the PSD complex (Tao-Cheng et al., 2015). The distance between the two vertical lines represents the length of island. The distance of the label was measured from the center of the particle to the outer edge of the plasma membrane (***C***, white arrows) for all the particles within the marked area. All islands in this figure were from soma. Scale bar, 0.1 µm.

NR islands are readily detectible due to the clustered gold particles (Fig. 1). Once we learned how to recognize the NR islands, islands could be identified based on the structural characteristics of the cytoplasmic density beneath the plasma membrane. Because not all islands label for all the antibodies, the percentage of island labeled was calculated for each antibody. Every neuronal somal profile encountered was scored for the presence of islands based on the characteristic dense material underneath the plasma membrane (Fig. 2*A*,*B*, arrows). Every island was photographed, regardless of whether the island was labeled for any particular antibody. The percentage of islands that were labeled from each experiment was then calculated for each antibody.

The labeling intensity of islands was calculated among labeled islands by counting the total number of particles within the marked area, then dividing by the length of the island, and was expressed as the number of particles/running micrometer of island (Fig. 1*C*). A ratio of labeling intensities between islands and PSDs within each experiment was calculated for each antibody. The laminar distributions of labels at islands were compared with those at the PSD. Because the laminar distribution of many of the PSD proteins was skewed, a nonparametric test (Wilcoxon test) was used to compare the medians. Finally, because somal and dendritic plasma membranes are continuous, it seemed reasonable to compare data gathered from somal islands with data from dendritic synapses.

## Results

### Distribution of NMDA receptors in dissociated hippocampal neurons

**Figure 3.**
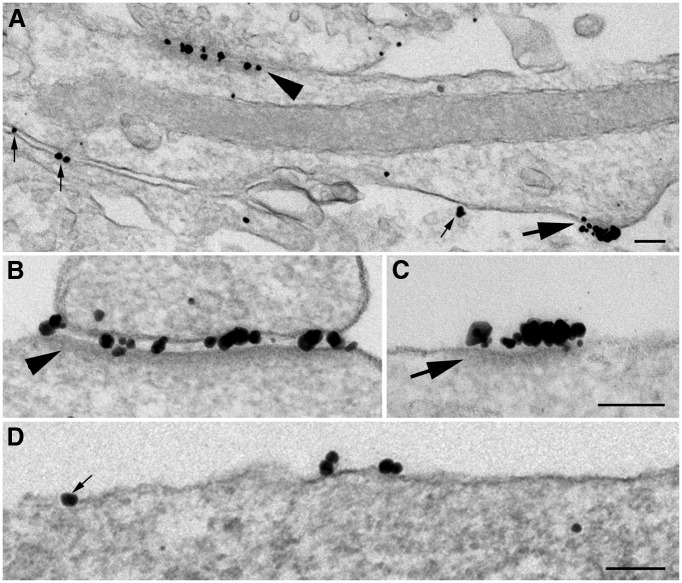
Distribution of NR2B on the plasma membrane of hippocampal neurons. Label for NR2B in 3-week-old dissociated hippocampal neurons prepared by pre-embedding immunogold labeling. ***A–D***, NR2B is concentrated at asymmetric synapses (***A***, arrowhead; ***B***, high magnification), but is also present in nonsynaptic parts of the plasma membrane, where it can be either in clusters (***A***, ***C***, large arrows) associated with a cytoplasmic density or as single individual grains (***A***, ***D***, small arrows). ***A*** and ***B*** were sampled from dendrites; ***C*** and ***D*** were sampled from soma. Scale bar, 0.1 µm.

Labeling patterns for NR1 and NR2B, two subunits of NMDA receptors that often coexist in a functional tetrameric complex (Wenthold et al., 2003), were similar in 3-week-old cultures. Because the NR2B antibody provided better labeling, most micrographs and all measurements are from samples labeled for NR2B. The following two structural entities stood out because of their high labeling intensities: asymmetric synapses (Fig. 3*A*,*B*, arrowheads); and nonsynaptic NR clusters (Fig. 3*A*,*C*, arrows). Both entities existed throughout somal and dendritic locations, and there was no structural distinction for either entity whether they were located on soma or dendrites. The synaptic distribution of NMDA receptors under different experimental conditions will be examined separately; here the focus is on the nonsynaptic NR clusters. We will refer to these clusters hereafter as nonsynaptic NR islands. These islands are additionally defined by a cytoplasmic density (Fig. 3*C*, arrow), which resembles a PSD (Fig. 3*B*, arrowhead).

The NR2B antibody was used here against an extracellular epitope, so particles representing silver-enhanced gold labels were located, as expected, in the synaptic cleft (Fig. 3*B*) or on the extracellular surface of the plasma membrane of an island (Fig. 3*C*). Scattered individual particles (Fig. 3*A*,*D*, small arrows) were also present along somal/dendritic plasma membranes at nonsynaptic locations, and the densities of these extrasynaptic labels were similar whether they were on somas or on primary dendrites extending from these somas. This observation is consistent with the observation from single-particle tracing using live light microscopy that extrasynaptic NMDA receptors are freely diffusible on plasma membranes (Groc et al., 2009).

Specificity of the NR2B antibody was also verified by comparing the labeling pattern on somal/dendritic plasma membrane with that on samples processed without primary antibody as well as with samples labeled with other antibodies, such as GKAP, Shank, and CAMKII. Compared to a labeling count for extrasynaptic NMDA receptors of 4.86 ± 0.41 labels per somal/dendritic profile (5 exp), there was negligible labeling on the extracellular side of the plasma membrane from these control samples (0.13, 0.05, 0.03, and 0.1 labels per somal/dendritic profile, respectively; *p* < 0.0001 in all cases, images not shown).

### Characterization of nonsynaptic NMDA receptor islands

**Figure 4.**
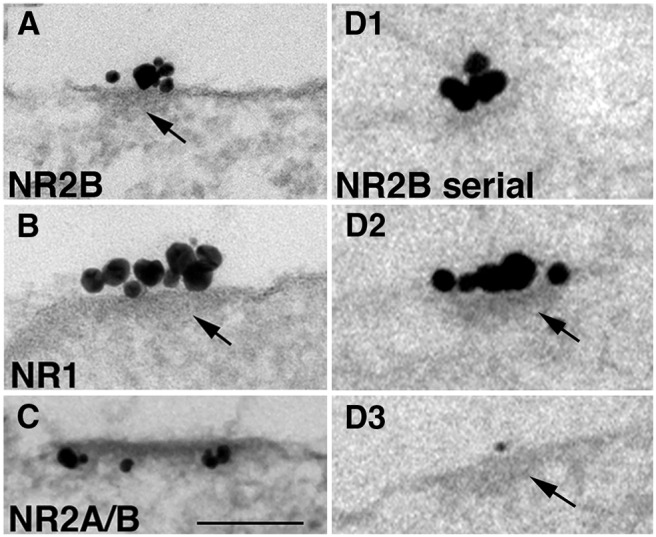
Nonsynaptic NR islands contain cytoplasmic densities (arrows) mimicking the structure of PSDs. ***A–C***, Islands label for different subunits of NMDA receptors as follows: NR2B in ***A***, NR1 in ***B***, and NR2A/B in ***C*** (the first two at extracellular epitopes). ***D1–D3***, Serial thin section analysis shows that labels are closely associated with an underlying density manifesting sharp borders. All islands in this figure were sampled from dendrites. Scale bar, 0.1 µm.

Islands consistently and prominently labeled with three different antibodies to the following NMDA receptors: NR2B (Fig. 4*A*); NR1 (Fig. 4*B*); and NR2A/B (Fig. 4*C*). Virtually all structurally identifiable islands showed tightly clustered labels. Islands viewed in single sections appeared to be heterogeneous in size (80-400 nm), with clusters of label for NR2B at densities of 20-80 labels/µm. Serial thin section analyses (Fig. 3*D1*–*D3*) from more than 50 islands (41 on soma, and 10 on dendrites) confirmed that there are no structural distinctions between somal and dendritic islands, that the label for NR is always closely associated with the underlying cytoplasmic densities (Fig. 4A, B, D2, D3, arrows), and that the label as well as the densities are limited by sharp borders.

### Overall concentration of NMDA receptors and the number of NR islands both increased after depolarization with high K^+^

**Figure 5.**
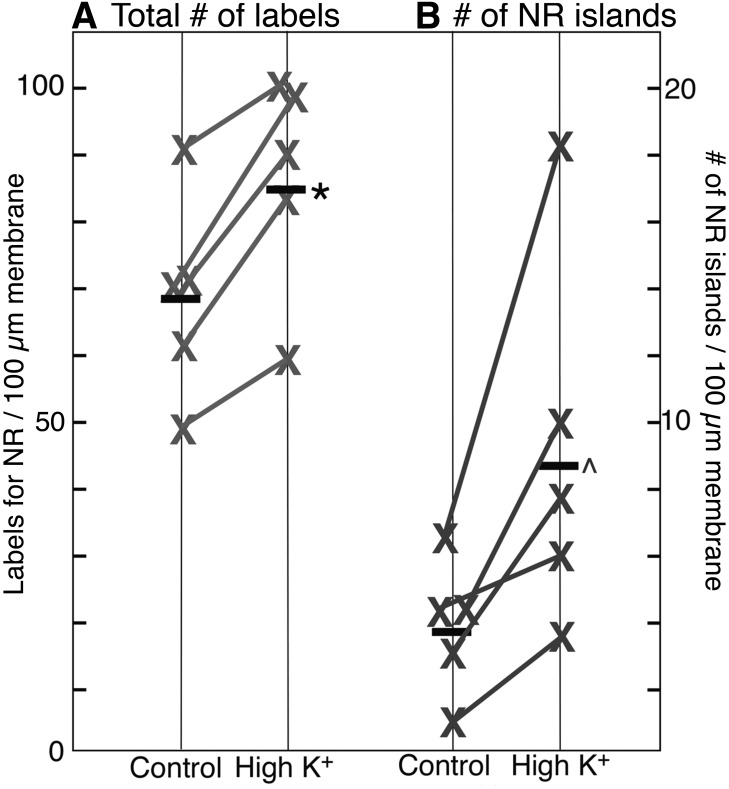
Labeling density of NMDA receptor and NR islands on somal plasma membrane after depolarization with high K^+^. ***A***, After high-K^+^ treatment (2 min, 90 mm), the overall labeling intensity for NR2B in somal plasma membranes increases to 126 ± 5% (*) of control values (five experiments, *p* < 0.01, paired *t* test). ***B***, After high-K^+^ treatment, the density of NR islands increases to 249 ± 34% (^) of control values (five experiments, *p* < 0.05, paired *t* test).

The overall labeling density of nonsynaptic NMDA receptors (number of gold particles per unit length of plasma membrane) on neuronal somas was measured in areas of the plasma membrane unopposed by processes of other cells, and counts included both individual particles and particles clustered in islands. The amount of label for receptors consistently increased after high K^+^ treatment (2 min, 90 mm) in five experiments (Fig. 5*A*), and the mean labeling density increased by 25%. This result suggests that additional receptors were inserted into the plasma membrane upon depolarization with high K^+^, and that these additional receptors were probably exocytosed onto the plasma membrane during depolarization.

The average number of NR islands per unit length of somal plasma membrane increased to 2.5-fold of controls after depolarization with high K^+^ (Fig. 5*B*), indicating that islands formed within 2 min upon depolarization. NR islands appear similarly induced to form in dendrites, as the number of NR islands per unit length of dendritic plasma membrane increased to approximately threefold of control values upon depolarization (2.48 ± 0.24 islands/100 µm dendritic plasma membrane in control vs. 7.23 ± 0.42 in high K^+^, 3 exp).

To test for the possibility that the epitope for NR antibody may have become more accessible upon depolarization, the numbers of islands were counted in identically treated samples labeled with other antibodies such as CaMKII, Shank, Homer, and GKAP. Because not all islands labeled for these other antibodies, islands were scored based on their structural characteristics (compare Fig. 2; Materials and Methods). The number of islands consistently increased upon high K^+^ treatment in 10 sets of samples (13.3 ± 1.9 islands/100 somas in control vs. 66.1 ± 6.6 in high K^+^ samples, *p* < 0.0001, paired *t* test).

It was interesting that the average length of the NR-labeled islands was smaller in high-K^+^ samples (130 ± 5 nm; range, 80-300 nm; *n* = 76) than in controls (175 ± 12 nm; range, 75-255 nm; *n* = 19; *p* < 0.001, paired *t* test, 5 exp). Also, there are many more small islands in the high K^+^-treated samples than in controls. These small islands cannot all be the result of breakdown subunits from larger islands because the sum of the lengths of all islands pooled from control samples was only ∼40% of that in high-K^+^ samples (3.32 µm from 89 soma for control samples, and 10.08 µm from 117 soma for high-K^+^ samples). Thus, there were indeed more small islands formed de novo after high-K^+^ treatment. These newly formed islands could result from direct insertion of a preformed cluster of receptors into the plasma membrane, or they might assemble quickly from individual receptors.

### NR islands are not exocytosed as a preformed package

**Figure 6.**
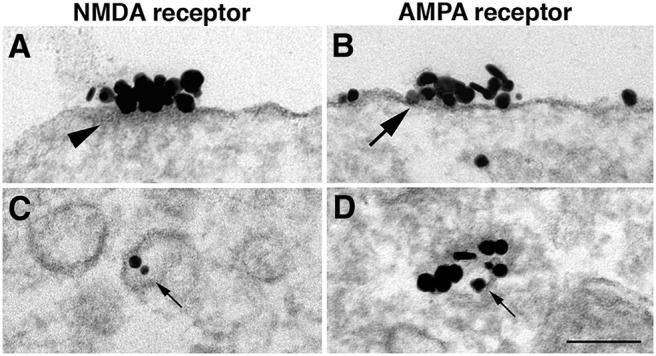
Comparison of NR islands and AMPA receptor patches. ***A***, ***B***, NR2B-labeled islands (***A***) have a distinct cytoplasmic density (arrowhead), which is lacking in AMPA receptor-labeled patches (***B***, GluR2 antibody). ***D***, AMPA receptor patches are thought to be exocytosed from cytoplasmic vacuoles containing concentrated receptors (small arrow). ***C***, In contrast, NR2B labels are typically at low concentrations, if present, in clear-membraned vacuoles (small arrows). ***A***, ***C***, and ***D*** were samples from dendrites, and ***B*** was from soma. Scale bars, 0.1 µm.

A search for evidence that islands are inserted into neuronal plasma membrane as a preformed package revealed no intracellular vacuoles containing concentrated NMDA receptors. Occasionally, vacuoles contained a few labels (Fig. 6*C*, small arrow), but these vacuoles never had associated cytoplasmic densities (Fig. 6*A*, arrowhead) and thus differed from the NR islands on the plasma membrane.

In contrast, patches of clustered AMPA receptors (Fig. 6*B*, large arrow) appear to be directly inserted into the plasma membrane as packages of concentrated receptors (Fig. 6*D*, small arrow; Tao-Cheng et al., 2011). These AMPA receptor patches (Fig. 6*B*) lack the cytoplasmic density that characterizes NR islands (Fig. 6*A*, arrowhead). Thus, AMPA receptor patches and NR islands are different structural entities.

### Dynamic disassembly of NR islands

To explore whether NR islands disassemble or persist after stimulation, cells were treated for 2 min with high K^+^ and then left to recover for 2-3 min or 30 min in control medium. The numbers of NR islands on somal plasma membrane increased significantly after 2 min of depolarization with high K^+^, quickly decreased to near control levels after 2-3 min of recovery, and stayed at that level after 30 min of recovery (Table 1). These results indicate that most NR islands disappear from surface membrane within 2-3 min of ending stimulation. Receptors clustered in islands could either scatter on plasma membrane as individual receptors or could be internalized as a package.

**Table 1:** Effects of stimulation on number of nonsynaptic NMDA receptor islands in neuronal plasma membranes

	Control	2 Min high K^+^	2 Min K^+^ + 2-3 min recovery	2 Min K^+^ + 30 min recovery
Exp 1	11.8 (17)	38.5 (26)	12.5 (40)	
Exp 2	13.0 (23)	35.0 (20)		16.0 (25)
Exp 3	10.3 (29)	68.1 (47)	18.5 (27)	18.2 (22)
Exp 4	5.0 (20)	57.7 (26)	11.5 (26)	16.2 (37)
Mean ± SEM	10.0 ± 1.8	49.8 ± 7.9*	14.2 ± 2.2	16.2 ± 0.7

Data are reported as number of islands per 100 somal profiles (n, Number of neuroanls somal profiles scored).

^*^The number of islands is significantly higher in high K^+^ vs. control (*P* < 0.0005), high K^+^ vs. 2 min K^+^+2-3 min recovery (*P* < 0.005) and high K^+^ vs. 2 min K^+^+30 min recovery (*P* < 0.005); ANOVA with Tukey’s post-test.

**Figure 7.**
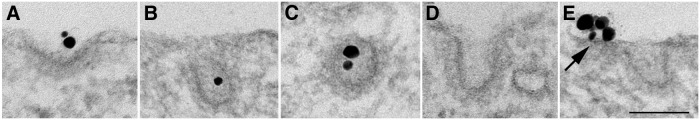
Endocytosis of NMDA receptors. ***A–C***, Label for NR2B is sometimes located in clathrin-coated pits (***A***, ***B***) and the lumens of coated vesicles (***C***). ***D***, ***E***, Many clathrin-coated pits are not labeled for NR2B. NR-labeled islands (***E***, arrow) may lie immediately adjacent to a coated pit, but are not endocytosed as an island. Scale bars, 0.1 µm.

In order to see whether NR islands are endocytosed as a package, we searched for vacuoles containing island-like cytoplasmic densities that could represent the aftermath of endocytosed islands. NMDA receptors are internalized by clathrin-mediated endocytosis (Roche et al., 2001; Nong et al., 2003; Petralia et al., 2003; Washbourne et al., 2004). Indeed, some clathrin-coated pits (Fig. 7*A*,*B*) and vesicles (Fig. 7*C*) contained a few labels for NR2B, but many clathrin-coated pits did not label for NMDA receptors (Fig. 7*D*,*E*). Occasionally, NR islands were present on plasma membranes immediately adjacent to coated pits (Fig. 7*E*, arrow), which is reminiscent of peri-PSD endocytic profiles (Petralia et al., 2003; Rácz et al., 2004), but islands with a cytoplasmic density were never present at a coated pit or vesicle.

### Nonsynaptic NMDA receptor islands contain many PSD proteins

**Figure 8.**
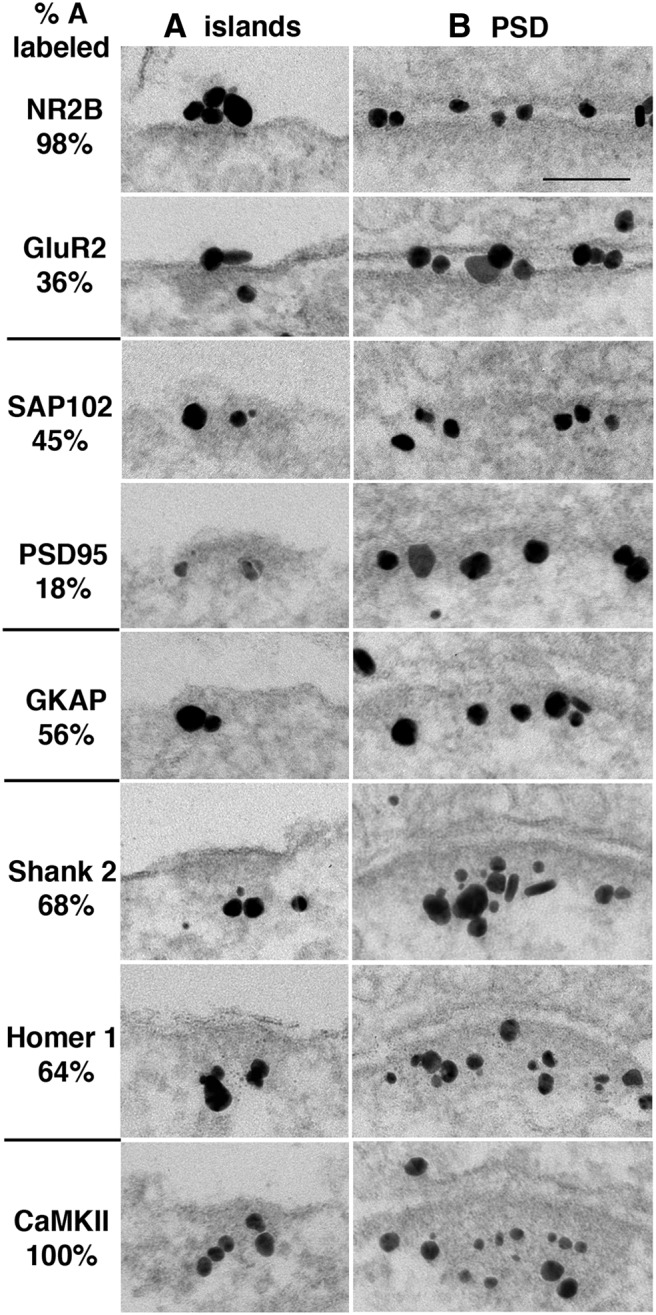
Islands and PSDs labeled for various PSD-associated proteins. Antibodies and the percentage of islands labeled are listed on the left. Images are arranged based on their laminar distribution at the PSD. Glutamate receptors at the top (NR2B and GluR2) are integral membrane proteins, here labeled with antibodies with extracellular epitopes. Extending successively deeper into the cytoplasm are the MAGUKs (SAP102 and PSD-95) immediately adjacent to the postsynaptic membrane; then GKAP, a binding partner of both PSD-95 and Shank; and a broad band of Shank and Homer, two scaffold proteins that bind to each other. Additionally, CaMKII, a kinase that can bind to NR2B or self-aggregate, is distributed throughout the PSD complex. The laminar localization of these proteins at islands mirrors that at PSDs. Images of islands and PSDs are all from the same sample, and samples are from experiments treated with 2 min high K^+^. All islands were sampled from soma, and all PSDs were sampled from dendrites. Scale bars, 0.1 µm.

Immunogold labeling of a set of proteins associated with PSDs was used to compare the composition of islands to that of PSDs. Because more islands appeared after treatment with high K^+^, we analyzed high K^+^-treated samples. Although virtually all islands labeled for NMDA receptors, not all islands labeled for all of the PSD proteins (Fig. 8).

Ranking from high to low in the percentage of islands labeled, NMDA receptors and CaMKII were detected in almost all islands. Shank, Homer, and GKAP were detected in 60-70%, GluR2 and SAP102 in 35-45%, and PSD-95 in only 18% of islands defined by structural criteria (Table 2). In contrast, virtually all PSDs in high K^+^-treated samples labeled for most PSD-associated proteins (Tao-Cheng et al., 2010; 2011; 2014; 2015) except for SAP102, which only labeled in about half of them.

**Table 2:** Percentage of NR islands labeled for PSD proteins and the ratios of labeling intensity at islands to that at PSDs

Antibodies	Percentage of islands labeled*^a^* (number of exp, number of islands scored)	Ratio of labeling intensity*^b^*
NR2B	97.5 ± 2.5 (4 exp, 42)	1.76 ± 0.13 (3 exp)
GluR2	36.3 ± 0.6 (2 exp, 33)	1.07 ± 0.24 (2 exp)
SAP102	44.7 ± 4.8 (6 exp, 113)	1.02 ± 0.15 (4 exp)
PSD-95	18.3 ± 4.8 (3 exp, 46)	0.39 ± 0.08 (3 exp)
GKAP	55.7 ± 15.9 (3 exp, 38)	0.70 ± 0.15 (3 exp)
Shank2	67.5 ± 12.5 (2 exp, 44)	0.60 ± 0.12 (2 exp)
Homer1	63.8 ± 6.4 (5 exp, 105)	0.59 ± 0.06 (3 exp)
CaMKII	100 (5 exp, 48)	1.0 ± 0.14 (3 exp)

Data are reported as the mean ± SEM.

*^a^*Percentage of islands labeled at neuronal somas in 3-week-old dissociated hippocampal cultures after depolarization with high K^+^. Islands were first identified by the structural characteristic of the cytoplasmic density, and then scored for presence of immunogold labeling with the various antibodies.

*^b^*Ratio of labeling intensities for each antibody as the number of labels per running micrometer of island divided by number of labels per running micrometer of PSDs in the same experiment.

### Differential distribution of PSD proteins at islands

Labeling intensities of PSD proteins at islands and PSDs were measured as number of labels per micrometer of island or PSD, and the ratio of labeling intensities of island to PSDs from the same sample was calculated for each antibody (Table 2). Numbers lower than 1 indicate that labeling intensity at islands is lower than that at PSDs.

NMDA receptors (NR2B) were much more prominent in islands than AMPA receptors (GluR2). Essentially, all islands labeled for NR2B, but only about one-third of islands labeled for the GluR2 subunit of AMPA receptors (*p* < 0.0001, *t* test). Between the two members of the membrane-associated guanylate kinase (MAGUK) family, SAP102 had a significantly higher presence at islands than PSD 95, both in the percentage of labeling (*p* < 0.01, *t* test) and in the ratio of labeling intensity (*p* < 0.05, *t* test).

Among the next three PSD scaffold proteins, GKAP, Shank, and Homer all showed similar labeling at islands that was consistently lower than that at PSDs, both in the percentage of islands labeled and in labeling intensity (Table 2). Interestingly, CaMKII had a strong presence at islands in high K^+^-treated samples where all islands labeled at the same intensity as that at PSDs (Table 2).

### Layered distributions of PSD proteins at islands are similar to those at PSDs

Distances of the label from the plasma membrane were measured to assess the laminar distribution of PSD proteins at islands. Measurements were taken from high K^+^-treated samples, where many more islands were present. Because some of the proteins (Shank2 and CaMKII) redistribute upon high K^+^ treatment, and the degree of redistribution is variable in different experiments, comparisons were made only within each experiment. Values for the median instead of the mean were used for a nonparametric statistical test between islands and PSDs because the distributions were typically skewed. There was no statistical difference in the distances of labels between islands and PSDs in any experiments (Table 3).

**Table 3:** Median distances of label to plasma membrane at islands and PSDs measured from high K^+^-treated samples

Antibody		Island (n, number of particles measured)	PSD (n, number of particles measured)
SAP102	Exp 1	26.7 (19)	26.7 (30)
	Exp 2	26.7 (17)	28.3 (18)
PSD-95	Exp 1	28.3 (14)*^a^*	26.7 (158)
	Exp 2		26.7 (130)
GKAP	Exp 1	35.0 (12)	33.3 (215)*^b^*
	Exp 2	30.0 (14)	30.0 (72)
Shank 2	Exp 1	56.7 (56)	50.0 (345)*^b^*
	Exp 2	43.3 (53)	46.6 (483)*^b^*
Homer 1	Exp 1	53.3 (49)	56.7 (120)
	Exp 2	50.0 (36)	53.3 (209)
CaMKII	Exp 1	53.3 (97)	53.3 (113)
	Exp 2	56.7 (84)	60.0 (135)

Data are reported as median distance in nanometer (nm).

*^a^*Pooled from 3 exp.

*^b^*Data are from the study by Tao-Cheng et al., 2015.

There was no statistical difference by Wilcoxon test between islands and PSDs.

Different proteins were localized within different layers at islands in an order similar to that at PSDs, as follows: SAP102, PSD-95, and GKAP were located in a narrow band close to the plasma membrane, while Shank, Homer, and CaMKII were in a broad band in the more distal part of the PSD complex (Fig. 7; Sans et al., 2000; Valtschanoff and Weinberg, 2001; Petralia et al., 2005; Yang et al., 2011; Tao-Cheng et al., 2014; 2015).

## Discussion

The present study uses pre-embedding immunogold electron microscopy to explicate the depolarization-induced redistribution of NRs, focusing on NR clusters at nonsynaptic locations. We refer to these structures as nonsynaptic NR islands and present evidence that they are not the immediate product of exocytosis of NMDA receptors, but form subsequent to the arrival of receptors on neuronal surfaces. Islands then incorporate PSD-associated proteins to eventually form a preassembled PSD-like entity. The formation and disassembly of islands provoked by depolarization is dynamic on a time scale of 2-3 min. This activity-dependent induction of NR islands provides a window during which nascent synapses could form after neuronal activity.

AMPA receptors tagged with pH-sensitive fluorescence probes are exocytosed in concentrated packages, giving rise to intense puffs of fluorescence when their acidic vesicular lumens are exposed to the neutral extracellular milieu (Yudowski et al., 2007). Similar exocytic events occur with other receptors, including transferrin receptors (Shen et al., 2014), but, so far, none have been reported for NMDA receptors (Groc et al., 2009). Electron microscopy verified the presence of intracellular vacuoles containing concentrated labels for AMPA and transferrin receptors (Tao-Cheng et al., 2011), but not for NMDA receptors. Thus, we speculate that NMDA receptors, unlike the AMPA receptors, may be exocytosed at low concentrations that are not readily detectible by direct, live, light microscopy via the pHfluorin method.

The differential recruitment of various PSD-associated proteins into islands mirrors that at developing synapses. NMDA receptors are much more prevalent than AMPA receptors at islands, which is consistent with early recruitment of NMDA receptors, and late arrival of AMPA receptors at both nascent synapses and nonsynaptic NR clusters (Rao et al., 1998; Petralia et al., 1999; Pickard et al., 2000; Washbourne et al., 2002; Shiraishi et al., 2003; Gerrow et al., 2006). All islands in high K^+^-treated samples labeled intensely for CaMKII, which is consistent with the early recruitment of CaMKII to PSDs during development (Swulius et al., 2010).

SAP102, a member of the MAGUK family, which includes PSD-95, is recruited to PSDs early during synapse development, later to be replaced by PSD-95 (Sans et al., 2000). Thus, developing PSDs, like islands, contain more SAP102 than PSD-95 (Petralia et al., 2010). Our results suggest that PSD-95 is incorporated into islands relatively late, which is consistent with observations by light microscopy at nascent synapses during PSD development (Washbourne et al., 2002; Barrow et al., 2009; Swulius et al., 2010).

GKAP, Shank, and Homer, three PSD scaffold proteins (Sheng and Kim, 2011), are present in ∼55-65% of NR islands, which is consistent with light microscopy observations that these proteins are at some, but not all, nonsynaptic NR clusters (Rao et al., 1998; Shiraishi et al., 2003; Gerrow et al., 2006). Although the labeling intensities of these scaffold proteins are lower at islands than at PSDs, their laminar distributions at islands are identical to those at PSDs. Altogether, our data indicate that NR islands contain a set of PSD proteins expected in nascent PSDs.

Preassembled specializations of presynaptic and postsynaptic proteins can form independently in early development, and both can initiate synapse formation (McAllister, 2007). Preformed PSD scaffold complexes are prevalent at nonsynaptic sites in young cells at 7 d *in vitro* (Gerrow et al., 2006), and some of them are mobile, but whether these complexes are at cell surfaces or are intracellular transport packets is unclear with light microscopy. By electron microscopy, it is clear that nonsynaptic NR islands reported here are at cell surfaces, but no intracellular entity representing preformed PSD complexes is evident in the 3-week-old cultures.

Nonsynaptic NR clusters, perhaps corresponding to the NR islands studied here, are common at surfaces of young cells by live cell imaging (Washbourne et al., 2004), and these may eventually be recruited to synaptic sites (Washbourne et al., 2002). Axonal processes occasionally contacted small clusters of NMDA receptors on dendrites, resulting in an entity resembling an incipient synapse. Alternatively, these axons may be retracting from incipient synapses, leaving the postsynaptic elements to become islands as remnants of transient contacts (Petralia et al., 2010).

Nonsynaptic NR islands form rapidly upon 2 min of depolarization with high K^+^. Because the measurements of number of islands were made on surfaces of neuronal somas facing the culture media, with no axons nearby, the increase in the number of islands cannot be caused by retracting axons. Although the high-K^+^ treatment is not physiological, it provides a convenient and consistent experimental level of stimulation for EM examination of structural changes at the synapses. Furthermore, depolarization with high K^+^ does not severely damage hippocampal neurons in culture, as cells recover fully (Dosemeci et al., 2001; Tao-Cheng et al., 2011).

Because islands can form on surfaces unapposed by other cellular processes, it is possible that the neuronal soma alone is capable of forming islands without direct interaction with other cellular elements. Alternatively, because high K^+^ likely induces the release of glutamate and/or other modulators from neurites and glia, islands might also be induced to form as a result of such releases from nearby processes. Regardless of the mechanism, 3-week-old hippocampal neurons are capable of forming islands rapidly upon induction. Whether this capability is age related awaits further investigation. Interestingly, islands are much more frequently seen in developing rats than in adults (Petralia et al., 2010), but whether islands are induced in developing brain via similar mechanisms as in neuronal cultures remains untested.

Our EM observations of 3-week-old neurons suggest that NMDA receptors are exocytosed and endocytosed as individuals or in small numbers. Once on the surface of the neuron, they cluster and become associated with other PSD proteins to form islands. Islands can be induced to assemble by depolarization of the neuron and spontaneously disassemble within minutes upon recovery. Within this short window, islands may be poised to attract nearby axons to form nascent synapses.

## Synthesis

The decision was a result of the Reviewing Editor Pablo Castillo and the peer reviewers coming together and discussing their recommendations until a consensus was reached. A fact-based synthesis statement explaining their decision and outlining what is needed to prepare a revision is listed below. The following reviewers agreed to reveal their identity: Ronald Petralia, Richard Weinberg

Your revised manuscript has been evaluated by three reviewers. All of them understand that your study provides good ultrastructural evidence for dynamic extrasynaptic NMDAR islands in neuronal cultures. However, the biological relevance of your findings remains unclear. The three reviewers also raised concerns with the way the manuscript has been assembled, and with the presentation and (over)interpretation of the data. Some important points raised by the reviewers are still unanswered and need attention.

MAJOR CONCERNS

1) Previous studies on synaptogenesis in neuronal cultures have investigated NMDAR clustering in dendrites. It is therefore important to know whether NR island formation upon depolarization by high K+ also occurs in dendrites. The authors should test this possibility as part of this study. In addition, they should examine whether the robust increase in NR islands leads to more synapses. If no changes are found, they should provide some explanation for their findings --or lack thereof-- and tone down all assertions regarding NR island formation as potential early step in synaptogenesis.

2) A more accurate definition for an NR island is required. As indicated in lines 123-126, "Nonsynaptic NMDA receptor islands are defined as patches of neuronal plasma membrane at nonsynaptic locations that label intensely for NMDA receptors and display characteristic [cytoplasmic] dense material." What is "label intensely"? Which of the used antibodies (NR2B, NR1, and NR2A/B; defines the blobs? How many islands/µm authors identify in the complete absence of antibodies? To help the reader understand what an NR island is about, Fig 1 could become Fig 1C (only); then Figure 2 would follow. The authors may want to include another low magnification image (possibly even lower than 2A) lacking immunostain, perhaps and/or a low-mag shot showing an obvious somatic profile.

3) The origin of the large increase in labeled NMDARs post-K+ should be further investigated. For example, the authors could test whether these NMDARs are newly synthesized and rapidly degraded by blocking protein synthesis or degradation, and then determining how those manipulations affect the increase in islands following high K+.

4) On pages 17-18, the authors state: "Thus, we speculate that NMDA receptors, unlike the AMPA receptors, may be exocytosed at low concentrations that are not readily detectible by live, light microscopy." They should make it clear that their result is specific for older cultures since this has in fact been demonstrated for NMDARs in young neurons using light microscopy in Washourne et al. 2004. Also, in the significance statement the authors state that "NMDA receptors, unlike AMPA receptors which are exocytosed in clusters, appear to be exocytosed only individually or in small groups. Islands must form by clustering of individual NMDA receptors already in the neuronal plasma membrane." Since it has been shown that NMDARs are exocytosed from transport packets in young neurons, the authors should make the distinction here that their results are from older cultures following massive depolarization. They might say, "in contrast to insertion of extrasynaptic NMDARs in young neurons..."

5) The writing needs attention/revision (see below).

SUGGESTED CHANGES TO THE TEXT:

#15, if the notion of synaptogenesis is intended, reword, as (for example) "...of 2-3 min, perhaps representing an initial stage of synaptogenesis."

#20, consider rewording as "NMDA receptors are thought to be exocytosed only..., so islands must form..."

#49 consider "..include cytoplasmic dense material resembling that..."

#51, suggestion: "...proteins, finding that many of these proteins localized to NR islands."

#57, replace "are" with "were."

#58 delete "furthermore."

#59, "was," not "is.". Consider deleting that whole sentence.

#101, should be "ethanols."

#135, "somas" please change throughout text to "somata."

#143, "somal" should be "somatic."

#145 "number of labels," this misuses the word "label." Perhaps "number of gold particles"? "number of black dots"?

#147, Fig 4A. The sequence of figures is confusing/confused. One expects figures to be numbered in sequence as presented in text. Authors may want to reorganize figure sequence.

#152-155, There are two versions of data presentation in the tables. Is there a reason to present as /100 µm in some places and as /100 somata in others? The former makes more sense.

#161, Most likely the authors mean to cite the table now listed "Table 3," though it would make more sense for this to in fact be Table 2.

#215, delete "again."

#216-217, what is meant by "disc-shaped"? Not perforated? not tortuous? or ?

#227-229, seems repetitious.

#229, "..total number of receptors" should be "...number of particles," or "amount of label."

#232-233 and several places later, an increase is an increase, but it would seem that additional receptor molecules don't have to be exocytosed. Couldn't they represent coalescence of very weak expression already present on nearby plasma membrane, or diffusion from dendritic membrane?

#238-241, text seems unduly wordy and clumsy. Consider: "to test for the possibility that the epitope for NR antibody may have become more accessible, ..."

#257-258, wordy and clumsy; this should be more directly and succinctly incorporated into the rest of the sentence.

#270-271. The observed difference in AMPAR patches from NMDAR patches is very interesting, but also slightly disturbing. On the one hand, this important result would benefit from better/clearer documentation to explain the basis of the result, even if only "it was our impression that...." On the other hand, it highlights the authors' seemingly fuzzy criteria for their NMDAR islands. In Table 3, they seem to have used patches of gold label as their primary criterion; if so, it would be interesting to know whether islands defined by other antigens do or do not associate with cytoplasmic densities. Table 3 also lists a substantial number of GluR2-positive "NR islands"; how does this reconcile with the result reported here?

#273-274; indeed, aftermath of exocytosis might be the basis, but is speculative, and if mentioned here there needs to be some kind of qualifier. Likewise, consider moving #275-280 into Discussion?

#284-285, consider rewording as follows: "To explore whether...after stimulation, we treated cells for 2 min...."

#295-309, the lack of clathrin-associated islands is worth reporting, but it is unclear whether NMDARs are necessarily endocytosed via clathrin, and their absence of evidence is only moderately strong as evidence of absence. Maybe they could simply tone the paragraph down a bit.

#330 etc., authors consider data from Table 3 column B before column A. If so, shouldn't they reverse the column sequence in the Table?

#359, Table 4 needs to be changed. Tao-Cheng et al. present their data in this format, but while authors may use these data to benefit, the data should be compressed to correspond to that in the other tables (not citing results on a 'by-experiment' basis).

#371, change "provides a window where."

#372-379, authors overdo their justification, both in length and number of repeats. Make a sensible case clearly and succinctly once, and be done with it.

#397-99, Delete? Sentence is true but probably not too important (since there's much more CaMKII than NR2B at synapses).

#403-405, if this idea is important enough to state, can it be worded more succinctly?

#412-415, please shorten.

#418, "initiated" remove the 'd.'

#421, consider a single sentence, "...are mobile, but whether...."

#423-426, reword/shorten as "..at cell surfaces; we detected no intracellular entity...cultures." (and delete the next sentence).

#427, a clarification should be included, e.g. "Nonsynaptic NR clusters (perhaps corresponding to the NR islands studied here) are common..."; this would allow deletion of the next sentence.

#431-433, consider rewording as "...an incipient synapse, though these axons may instead be retracting from incipient synapses, leaving the..."

#434-438, Delete?

#445, put a comma between "culture" and "as cells."

#449 "form" should be "from."

#449-451, glutamate is just one of several possibilities. Is there any reason to focus on glutamate?

Tables. In "combined" results, do authors treat each "experiment" as a data point? Please clarify as numbers do not seem to add up (e.g. duplicating "Control" column of Table 1 renders slightly different numbers than those provided by the authors). Authors should better explain (perhaps in Methods) what is meant by an "experiment." Also, in Table 1, authors list # of islands vs antibody; but it sounds as though either authors are double-labeling, or are identifying islands based on presence of proteins other than the NMDA receptor, seemingly at variance with their definition. Similar concerns apply to Table 3.
